# An Account of Two Cases of Lithotomy, Where the Wounds Were Healed by the First Intention

**Published:** 1799-10

**Authors:** Griffith Rowlands

**Affiliations:** Member of the Corporation of Surgeons, London, Surgeon to the Infirmary, and to the Lying-in Charity in Chester.


### *An Account of two Cases of Lithotomy, where the wounds were healed by the first Intention.* By GRIFFITH ROWLANDS, *Member of the Corporation of Surgeons, London, Surgeon to the Infirmary, and to the Lying-in Charity in Chester.*

JOHN GOLATHAN, aged four years and eight months, admitted into the Chester Infirmary on June the 5th, 1787, was cut for the stone on the eighth, and discharged cured, on the 19th of the same month.

The lips of the wound were brought together with slips of sticking-plaster and dressed with ceratum spermaceti spread on lint; a compress of linen (sparingly moistened with spirits of wine *) was then applied, and retained with the T bandage. I took particular care in the application of the bandage, to make it support the lip of the wound next the raphé. My patient was placed in bed on his *right side*, a slip of old linen tied round his knees to keep them together, and pinned to the sheet, to prevent his turning on his back. He took eight drops of laudanum immediately after he was dressed, and very soon fell into a sound sleep; but this being my first attempt, after the operation of lithotomy, to deny the urine a passage through the wound, I was very watchful over him. I determined, if he did not pass his urine through the penis in four hours, to remove the dressings; but at the end of three hours, he made water freely, and went to sleep again, without disturbing the wound. I did not dress him until the 12th, when

* In every case where it is my wish to unite wounds by the first intention, as after amputation, I always moisten the compress and external covering with spirit of wine or brandy, from which I have derived much advantage.

when I found the lips of the wound completely united, and on the 19th he quitted the Infirmary in perfect health.

On the 19th of September, 1795, I cut THOMAS SORTON, a child of three years of age, and took from him a ftone weighing two drachms—he was treated in the fame manner as Golathan, in regard to dreffing and pofition in bed, and recovered without any interruption. He left the Infirmary in feventeen days from the operation.

Every experienced practitioner muft be aware, that this plan can only anfwer in cafes where the ftone is eafily extracted, and where there is a certainty that no fragments are left behind. I think, likewife, that it is more likely to fucceed in children than adults, from the proportionable fmallnefs of the wound, &c. &c.

